# Clinically Suspected Neutropenic Enterocolitis Before Documented Neutropenia in a Patient With Breast Cancer: A Case Report

**DOI:** 10.7759/cureus.117948

**Published:** 2026-10-14

**Authors:** Gayathri Sadanandan, Hnin Wai Yan

**Affiliations:** 1 Department of Acute and General Medicine, East Lancashire Hospitals NHS Trust, Blackburn, GBR

**Keywords:** breast cancer, docetaxel, neutropenia, neutropenic enterocolitis, typhlitis

## Abstract

Neutropenic enterocolitis (NE), or typhlitis, is a potentially life-threatening complication of cytotoxic chemotherapy classically characterized by neutropenia, fever, and abdominal pain. However, these features may not develop simultaneously, creating a diagnostic challenge when gastrointestinal symptoms precede documented neutropenia.

A 44-year-old woman with invasive ductal carcinoma of the breast presented five days after her fourth overall cycle of adjuvant chemotherapy, documented as docetaxel treatment, with worsening abdominal pain, vomiting, rigors, and constipation. Initial laboratory testing demonstrated an absolute neutrophil count (ANC) of 2.3 × 10⁹/L and a C-reactive protein (CRP) of 20 mg/L, confirming that she was not neutropenic. An initial abdominal radiograph showed fecal loading, providing a plausible early explanation for her symptoms. Within 24 hours, the patient developed pyrexia and profound neutropenia with an ANC of 0.1 × 10⁹/L and CRP rising to 133 mg/L. Subsequent review of the initial abdominal radiograph identified a featureless descending colon with loss of normal haustral markings, raising concern for colitis. She was managed conservatively with intravenous broad-spectrum antimicrobials, fluid support, filgrastim, cautious bowel management, and multidisciplinary review. She achieved clinical improvement and neutrophil recovery without surgical intervention and was discharged on hospital Day 7.

This case suggests that gastrointestinal symptoms concerning for NE may precede documented neutropenia. A normal initial neutrophil count should therefore not provide false reassurance when gastrointestinal symptoms develop following chemotherapy and subsequently persist or progress. Serial clinical assessment, repeat hematological testing, appropriate imaging, radiological reassessment, and multidisciplinary review are important when the clinical course evolves.

## Introduction

Neutropenic enterocolitis (NE), historically termed typhlitis, is a potentially life-threatening gastrointestinal complication most commonly encountered in patients with severe neutropenia following cytotoxic chemotherapy. Although traditionally associated with hematological malignancies, NE has also been reported in patients receiving chemotherapy for solid tumors, including breast cancer. Its pathogenesis is considered multifactorial, involving chemotherapy-induced mucosal injury, impaired host defense associated with neutropenia, and microbial translocation across the damaged intestinal mucosa [1,2]. Severe disease may progress to gastrointestinal bleeding, bowel necrosis, perforation, sepsis, and death [1,2].

The clinical presentation of NE is variable. Abdominal pain, fever, neutropenia, and diarrhea are commonly described; however, these features may not occur simultaneously, and involvement is not invariably confined to the ileocecal region [1,2]. This variability can make early recognition challenging, particularly when gastrointestinal symptoms develop before profound neutropenia is documented. The differential diagnosis in patients receiving chemotherapy is broad and includes treatment-related gastrointestinal toxicity, infectious colitis, bowel obstruction, bowel ischemia, and other acute intra-abdominal pathology.

Imaging forms an important component of the diagnostic assessment. Computed tomography (CT) can demonstrate bowel-wall thickening, pericolic inflammatory change, bowel dilation, pneumatosis, and complications such as perforation [1,2]. Plain abdominal radiography is less sensitive and may demonstrate nonspecific abnormalities, including bowel dilation, ileus, pneumatosis, or free intraperitoneal gas [1,2]. Diagnosis therefore requires interpretation of imaging findings alongside the clinical course, hematological parameters, and alternative diagnoses. Management is predominantly conservative in uncomplicated cases and may include bowel rest, fluid and electrolyte support, and broad-spectrum antimicrobial therapy, with granulocyte colony-stimulating factor (G-CSF) considered in selected patients [1,2]. Invasive or potentially traumatic bowel interventions require particular caution in suspected NE because of the risk of bowel-wall injury and perforation [1,2].

Published evidence regarding NE specifically in patients with breast cancer remains limited and consists predominantly of case reports and small series. Reported cases describe variable clinical presentations and potentially serious complications, highlighting the importance of examining this uncommon complication in the breast cancer population [3-18].

We present the case of a 44-year-old woman receiving adjuvant chemotherapy for breast cancer who developed abdominal pain and constipation while initially nonneutropenic, with initial plain abdominal radiography demonstrating fecal loading. Within 24 hours, she developed profound neutropenia and systemic inflammatory deterioration, while subsequent review of the initial radiograph raised concern for colitis. This case highlights the diagnostic challenge posed by gastrointestinal symptoms preceding documented neutropenia and the importance of reassessment when the clinical course evolves following chemotherapy.

## Case presentation

A 44-year-old woman with left breast invasive ductal carcinoma (IDC) presented to the emergency department five days after her fourth overall cycle of adjuvant chemotherapy with a two-day history of worsening umbilical abdominal pain, vomiting, rigors, and intermittent pleuritic chest pain radiating to the upper back. Her planned treatment was a six-cycle EC-docetaxel regimen comprising epirubicin, cyclophosphamide, and docetaxel, administered at three-week intervals. The fourth cycle was documented as docetaxel treatment, with prophylactic filgrastim (600 μg) prescribed.

She had been diagnosed in June 2025 and underwent left wide local excision and sentinel lymph node biopsy in August 2025. Pathology demonstrated a 35-mm, grade 3 IDC with associated high-grade ductal carcinoma in situ, a total disease extent of 55 mm, and no lymph node involvement (0/2; pT2 pN0). The tumor was estrogen receptor-positive, progesterone receptor-positive, and human epidermal growth factor receptor 2-negative. Her medical history included possible previous hypertension; she took no regular medications and had no known drug allergies.

Given her recent chemotherapy and presenting rigors, she was triaged in the emergency department for suspected neutropenic sepsis, and the local neutropenic sepsis protocol was activated. Initial vital signs showed a temperature of 37.0°C, heart rate 93 beats/minute, blood pressure 149/85 mmHg, respiratory rate 18 breaths/minute, and oxygen saturation 100% on room air. Blood cultures and laboratory investigations were obtained, and she received intravenous fluids and an initial empiric dose of intravenous piperacillin-tazobactam. A chest radiograph and electrocardiogram were also performed prior to referral to the Same Day Emergency Care (SDEC) unit for further assessment.

Initial blood tests demonstrated a white blood cell count of 3.4 × 10⁹/L, an absolute neutrophil count (ANC) of 2.3 × 10⁹/L, and a C-reactive protein (CRP) level of 20 mg/L, confirming that she was not neutropenic at initial presentation. Her D-dimer was elevated at 1,630 ng/mL, compared with 1,437 ng/mL recorded 10 days prior. This previous elevation had been investigated with right upper limb Doppler ultrasonography to exclude peripherally inserted central catheter (PICC)-associated thrombosis, which demonstrated no evidence of deep-vein thrombosis. Serial laboratory investigations during admission are summarized in Table 1.

**Table 1:** Serial laboratory investigations during admission

Parameter	Reference range	Day 1 (initial presentation)	Day 2	Day 3	Day 4	Day 7 (discharge)
White blood cell count (×10⁹/L)	4.0-11.0	3.4	0.4	4.8	34.5	31.1
Absolute neutrophil count (×10⁹/L)	2.0-7.5	2.3	0.1	2.9	29.0	27.4
C-reactive protein (mg/L)	<3.3	20	133	232	177	37
Hemoglobin (g/L)	115-165	119	118	113	110	101
D-dimer (ng/mL)	0-500	1,630	-	-	-	-

Upon examination in SDEC, she exhibited localized epigastric and left upper quadrant tenderness in the setting of severe constipation. Plain abdominal radiography demonstrated marked fecal loading without overt bowel dilation (Figure 1).

**Figure 1:**
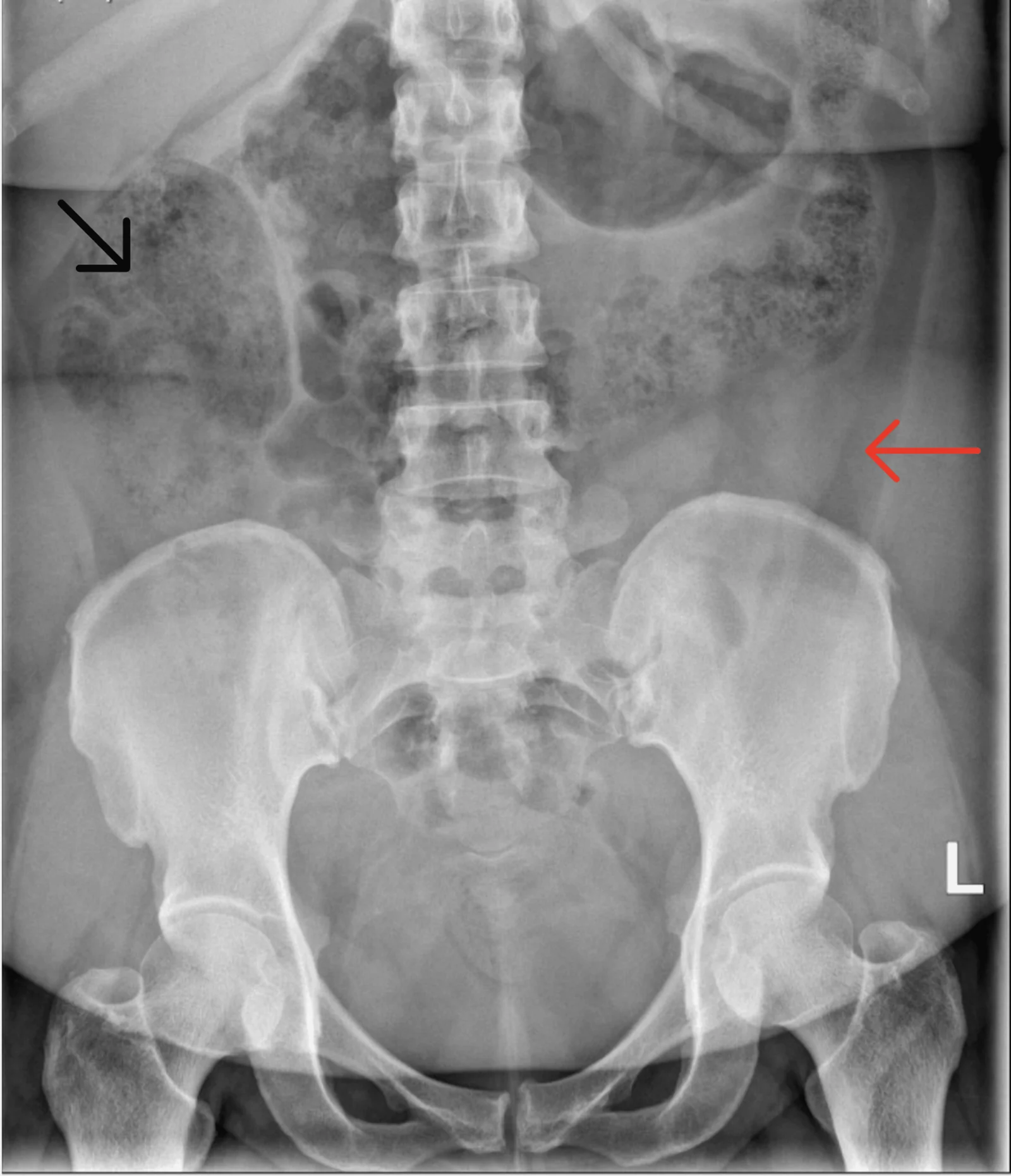
Plain abdominal radiograph demonstrating marked fecal loading in the ascending colon (black arrow) and a featureless appearance of the descending colon with loss of normal haustral markings (red arrow), raising concern for colitis.

Initial working differential diagnoses included chemotherapy-induced gastrointestinal toxicity, constipation, and acute gastritis. On Day 1, conservative management commenced with oral laxatives (macrogol, one sachet daily) and a phosphate enema, before profound neutropenia was documented. On Day 2 of admission, the patient developed an overnight pyrexial spike. Repeat laboratory testing demonstrated profound neutropenia and a marked rise in CRP (Table 1). A peripheral blood film revealed marked neutropenia and red blood cell anisocytosis without circulating blasts, consistent with chemotherapy-induced myelosuppression.

The patient was formally admitted and managed for neutropenic sepsis. Empiric broad-spectrum antimicrobial therapy with intravenous piperacillin-tazobactam was given alongside G-CSF (filgrastim, two total doses administered on Days 2 and 3) to promote neutrophil recovery. Despite the identification of profound neutropenia on Day 2, two Micralax enemas (Meribel Pharma Karlskoga AB, Sweden) were administered; the clinical rationale for their continued use was not documented. On Day 3, the ward-round assessment recorded persistent abdominal pain, bloating, and inadequate bowel movements over the preceding five days, and a further phosphate enema was prescribed as part of the management plan. Oral laxatives were titrated sequentially up to three sachets daily. Oral nystatin and potassium replacement were also administered during admission. Following bowel evacuation, the patient reported temporary alleviation of abdominal cramps.

Subsequent review of the initial abdominal radiographs demonstrated fecal loading in the ascending and transverse colon alongside a featureless, ahaustral appearance of the descending colon without luminal feces (Figure 1), raising concern for acute colitis. CT of the abdomen and pelvis was requested, but following discussion with radiology to review the request, the radiologist considered the abdominal radiographic findings suggestive of colitis and advised that CT would not provide additional diagnostic information if neutropenic colitis was clinically suspected. CT was therefore not performed.

Later on Day 3, after the final phosphate enema, gastroenterology advised cautious laxative use, adjusting oral macrogol to two to three sachets daily, and avoiding further rectal enemas due to concern for bowel-wall injury and perforation. Stool cultures and *Clostridioides difficile* toxin assays were sent.

Microbiology consultation was obtained regarding antimicrobial strategy and duration. Microbiology advised that, because neutropenic colitis can arise secondary to noninfectious mucosal injury or viral mucosal barrier breach, a five-day course of intravenous piperacillin-tazobactam was deemed clinically sufficient in the event of possible transient bacteremia, provided clinical stability was maintained. Paired blood cultures obtained approximately 24 hours after antibiotic initiation remained negative; therefore, bacteremia was not microbiologically confirmed.

A serial blood film examination on Day 4 demonstrated rapid granulocytic recovery with marked leukocytosis and neutrophilia (Table 1), alongside a marked left shift with toxic granulation, myelocytes, metamyelocytes, nucleated red blood cells, and monocytosis, reflecting a robust marrow response to G-CSF.

A comprehensive infectious workup returned negative across all modalities. Sequential peripheral and PICC line blood cultures drawn across Days 1-4 demonstrated no growth. Stool cultures and *C. difficile* toxins A/B were negative; a respiratory viral polymerase chain reaction panel (COVID-19, Influenza A/B, respiratory syncytial virus) showed no viral pathogens; and midstream urine cultures showed no growth.

Following neutrophil recovery and sustained afebrility, the patient demonstrated complete clinical recovery. Inflammatory markers improved significantly, with CRP falling to 37 mg/L, while leukocytosis and neutrophilia persisted following G-CSF administration (Table 1). The patient was successfully discharged on hospital Day 7 with a final diagnosis of neutropenic sepsis, with colitis considered as the possible source.

She was discharged on oral co-amoxiclav to complete a total 10-day antibiotic course, alongside maintenance macrogol (one sachet twice daily).

At oncology follow-up two weeks later, options for subsequent chemotherapy included weekly paclitaxel or dose-reduced three-weekly docetaxel. The patient opted for dose-reduced three-weekly docetaxel and subsequently completed all six planned cycles of chemotherapy with no documented recurrence of similar gastrointestinal symptoms. She subsequently completed adjuvant radiotherapy and commenced adjuvant endocrine therapy with letrozole and ovarian suppression, alongside ribociclib. At the most recent oncology review, she was tolerating treatment without significant reported toxicity.

## Discussion

This case highlights the diagnostic challenge of clinically suspected NE when gastrointestinal symptoms precede documented profound neutropenia. The patient presented five days following her fourth cycle of adjuvant chemotherapy with abdominal pain and constipation but was initially nonneutropenic, with an ANC of 2.3 × 10⁹/L. Plain abdominal radiography demonstrated fecal loading, providing a plausible initial explanation for her symptoms. However, within 24 hours, she developed pyrexia, profound neutropenia with an ANC of 0.1 × 10⁹/L, and a marked systemic inflammatory response, with CRP rising from 20 to 133 mg/L. Subsequent review of the initial abdominal radiograph identified additional features concerning for colitis. The evolving clinical, hematological, and radiographic picture therefore prompted reconsideration of the initial diagnosis and underscores the importance of ongoing clinical reassessment in patients who develop gastrointestinal symptoms following myelosuppressive chemotherapy.

A focused literature search was conducted in PubMed using the search terms “neutropenic enterocolitis”, “breast cancer”, and “chemotherapy”. Relevant publications were reviewed, and cases involving patients with breast cancer were selected when sufficient individual patient information was available to assess clinical presentation, chemotherapy exposure, and outcomes. Eighteen eligible patient-level cases were included in the descriptive review. The taxane-associated colitis cohort reported by Li et al. was considered separately and was not included in the 18-case denominator.

NE classically occurs in the setting of neutropenia, fever, and abdominal pain, although these features may not develop simultaneously [1,2]. Clinical variability was similarly observed in our review of 18 published patient-level breast cancer cases, five of whom (27.8%) developed gastrointestinal symptoms before neutropenia was documented [3-18].

Additional evidence for variability in presentation is provided by Li et al., who reported a cohort of 14 patients with breast cancer who experienced 16 episodes of colitis following taxane-based chemotherapy [19]. While abdominal pain was present in all 16 episodes, constipation was documented in four (25.0%). Notably, eight episodes occurred with both neutropenia and fever, whereas six occurred in the absence of both. The median onset was six days following chemotherapy (range: three to eight days). In our patient, gastrointestinal symptoms began approximately three days after chemotherapy, with presentation occurring on Day 5. These findings further demonstrate that taxane-associated colitis may present without the simultaneous presence of neutropenia and fever and that constipation may form part of the initial gastrointestinal presentation [19]. Importantly, the Li et al. cohort describes taxane-associated colitis more broadly, and episodes occurring without neutropenia should not be interpreted as confirmed NE.

Taxane exposure was common among the published breast cancer cases identified in our review (Table 2). Of the 18 eligible patient-level cases, 12 (66.7%) occurred following taxane-containing regimens, including 11 involving docetaxel and one involving paclitaxel [3-18]. Among the 13 cases in which treatment-cycle information was available, 10 (76.9%) occurred following the first cycle of the reported treatment regimen [3-18]. In the present case, gastrointestinal symptoms developed five days after the fourth overall chemotherapy cycle, which was documented as docetaxel treatment. The available records did not establish whether this represented the patient's first exposure to docetaxel, limiting direct comparison with first-cycle events in published reports. Li et al. similarly reported taxane-associated colitis following both docetaxel and paclitaxel-based chemotherapy [19]. However, the predominance of taxane exposure among published cases should not be interpreted as establishing causality or comparative treatment risk, given the small number of cases and the potential for reporting and publication bias.

**Table 2:** Summary of 18 published patient-level cases of neutropenic enterocolitis in patients with breast cancer Treatment-cycle information was available for 13 of the 18 cases. Categories were not necessarily mutually exclusive CT: computed tomography; G-CSF: granulocyte colony-stimulating factor

Characteristic	Cases reported	Percentage (%)
Chemotherapy exposure		
Taxane-containing chemotherapy	12 out of 18	66.7
Docetaxel exposure	11 out of 18	61.1
Paclitaxel exposure	1 out of 18	5.6
First cycle of the reported treatment regimen	10 out of 13	76.9
Clinical presentation		
Gastrointestinal symptoms before documented neutropenia	5 out of 18	27.8
Imaging		
CT imaging	14 out of 18	77.8
Abdominal radiography	4 out of 18	22.2
Management		
Antibiotic treatment	17 out of 18	94.4
G-CSF treatment	7 out of 18	38.9
Surgical intervention	8 out of 18	44.4
Outcomes		
Bowel perforation	5 out of 18	27.8
Death	6 out of 18	33.3

Imaging plays a pivotal role in evaluating suspected NE. CT is more sensitive than plain abdominal radiography and may demonstrate bowel wall thickening, pericolic inflammatory changes, bowel dilation, pneumatosis, and complications such as perforation [1,2]. In the cohort reported by Li et al., CT was abnormal in all 10 patients who underwent the modality, whereas plain abdominal radiography demonstrated abnormalities in three of the nine patients evaluated [19]. In the present case, the initial abdominal radiograph demonstrated fecal loading; however, subsequent radiological review identified a featureless appearance of the descending colon with loss of normal haustration, raising concern for colitis. Although CT of the abdomen and pelvis was requested, it was not performed following radiological advice that it would not add further diagnostic information in the presence of clinical suspicion and radiographic findings suggestive of colitis. Nevertheless, the absence of cross-sectional imaging limited diagnostic certainty and assessment for complications, and the radiographic findings alone could not confirm NE.

Constipation was a prominent feature of the patient's initial presentation and, combined with the fecal loading demonstrated on abdominal radiography, offered a plausible explanation for her abdominal pain. Bowel-directed treatment with macrogol and enemas was initially commenced for constipation and radiographic fecal loading before profound neutropenia was documented. However, rectal enemas continued on Days 2 and 3 despite documented neutropenia and evolving concern for possible typhlitis. The Day 3 ward-round assessment recorded persistent pain, bloating, and inadequate bowel movements as the context for the final enema prescription. Gastroenterology subsequently advised avoiding further rectal enemas because of concern regarding bowel-wall injury and perforation. This is particularly relevant in NE, in which mucosal injury and bowel-wall inflammation may predispose to serious complications, and potentially traumatic bowel interventions should therefore be approached cautiously [1,2]. Constipation itself may also occur in taxane-associated colitis; Li et al. reported constipation in four of 16 episodes (25.0%) [19].

Management of NE remains predominantly conservative without surgical complications, focusing on supportive care and broad-spectrum antimicrobial therapy [1,2]. In our review, antibiotic therapy was reported in 17 of 18 cases, and therapeutic G-CSF was noted in seven [3-18]. Eight patients ultimately required surgical intervention, most commonly right-sided colonic resection [3-18]. In the present case, the patient was successfully managed conservatively with intravenous piperacillin-tazobactam, fluid support, filgrastim, and close multidisciplinary oversight involving oncology, gastroenterology, radiology, and microbiology. Subsequent clinical improvement and neutrophil recovery precluded the need for surgical intervention.

Despite predominantly conservative initial management, NE can be associated with serious complications. In our review of 18 published patient-level breast cancer cases, bowel perforation was reported in five patients (27.8%), and six patients (33.3%) died [3-18]. Similarly, Li et al. reported that two patients suffered bowel perforation secondary to transmural necrosis, both requiring hemicolectomy. Separately, one patient succumbed to septic shock, with blood cultures growing *Stenotrophomonas maltophilia* and coagulase-negative Staphylococcus [19]. These findings underscore the potential severity of this condition and the importance of early recognition alongside vigilant clinical monitoring. However, these figures should not be interpreted as definitive estimates of perforation incidence or overall mortality in NE, given that the current literature consists primarily of isolated case reports and small series that are inherently vulnerable to publication and reporting bias.

A limitation of the present report is the absence of cross-sectional imaging to characterize bowel-wall inflammation and assess for complications. Consequently, the formal discharge diagnosis was recorded as neutropenic sepsis, with colitis considered as the possible source. Alternative explanations for the gastrointestinal symptoms include chemotherapy-related gastrointestinal toxicity, severe constipation, and infectious colitis. The initial fecal loading supported constipation as a possible contributor, while the subsequent fever, profound neutropenia, and inflammatory deterioration raised concern for a more serious underlying process. Negative microbiological investigations did not establish a specific infectious cause, and the absence of cross-sectional imaging prevented definitive characterization of the suspected colonic inflammation. Nevertheless, clinical suspicion of NE was supported by the temporal relationship with chemotherapy, acute gastrointestinal symptoms, subsequent development of profound neutropenia and pyrexia, rising inflammatory markers, and plain radiographic features concerning for colitis. This case should therefore be interpreted as clinically suspected NE rather than a radiologically or histologically confirmed entity. This diagnostic uncertainty must be considered alongside the inherent limitations of the published literature, which relies primarily on isolated case reports and small series characterized by heterogeneous diagnostic approaches and incomplete reporting.

The principal clinical implication is that persistent or worsening gastrointestinal symptoms following chemotherapy warrant reassessment, even when initial investigations appear reassuring. Given the diagnostic uncertainty in this case, timely cross-sectional imaging, where clinically appropriate, and multidisciplinary evaluation remain important for distinguishing suspected NE from alternative causes of acute abdominal symptoms.

## Conclusions

Gastrointestinal symptoms concerning for clinically suspected NE (typhlitis) may precede documented profound neutropenia, creating a diagnostic challenge in patients receiving chemotherapy. Although constipation and fecal loading provided a plausible initial explanation for this patient’s abdominal pain, subsequent pyrexia, profound neutropenia, rising inflammatory markers, and radiographic features concerning for colitis prompted reconsideration of the initial diagnosis.

This case emphasizes the importance of maintaining a high index of suspicion for NE when gastrointestinal symptoms develop following chemotherapy, even when the initial ANC is within normal limits. Serial clinical assessment, repeat hematological evaluation, appropriate imaging, and prompt multidisciplinary review when the clinical course evolves may facilitate timely recognition, appropriate management, and early identification of potentially life-threatening complications.
